# Dual inhibition of epidermal growth factor and insulin-like 1 growth factor receptors reduce intestinal adenoma burden in the *Apc*^*min*/+^ mouse

**DOI:** 10.1038/bjc.2011.291

**Published:** 2011-08-02

**Authors:** P H S Shaw, T S Maughan, A R Clarke

**Affiliations:** 1 School of Bioscience, Cardiff University, Museum Avenue, Cardiff CF10 3AX, Wales, UK; 2 MRC/CR-UK Gray Institute for Radiation Oncology and Biology, Department of Oncology, University of Oxford, Old Road Campus Research Buildings, Off Roosevelt Drive, Churchill Hospital, Oxford OX3 7DQ, UK

**Keywords:** epidermal growth factor receptor, insulin-like 1 growth factor receptor, receptor tyrosine kinase inhibition, *Apc*^*min*/+^ mouse

## Abstract

**Background:**

Identification of early molecular pathway changes may be useful as biomarkers for tumour response/resistance prediction, and here we provide direct *in vivo* proof of this concept. The type 1 insulin-like growth factor receptor (IGF1R) has been implicated in various aspects of adenoma development and metastasis. We show here that, in murine intestinal adenomas acutely exposed to a small molecular inhibitor of EGFR (gefitinib), there is concurrent suppression of EGFR downstream signalling and induction of IGF signalling. We therefore tested the hypothesis that blockade of EGFR signalling was being tempered by compensatory activation of the IGF pathway by examining the effect of chronic suppression of IGF1R using AZ12253801, a small molecular tyrosine kinase inhibitor of IGF1R.

**Methods:**

Male *Apc*^*min*/+^ mice with an intestinal tumour burden were exposed to a single dose of an inhibitor against EGFR (gefitinib), IGF1R (AZ12253801), 0.5% Tween 80 or combined EGFR/IGF1R inhibitor and culled 4 h post dosing. Tumour tissue was analysed to detect the early molecular pathways induced and anti-tumour phenotypic changes. Cohorts of male *Apc*^*min*/+^ mice (*n*=15–17) were subsequently treated with gefitinib for a period of 8 weeks and subsequently exposed to single (either gefitinib or AZ12253801) or combined (gefitinib and AZ12253801) therapy. We also included a vehicle-treated cohort, which was never exposed to gefitinib and became symptomatic of the disease by day 150.

**Results:**

Both single treatments delayed the onset of disease symptoms. Combined dosing with gefitinib and AZ12253801 similarly delayed the onset of symptoms, and at 200 days suppressed small intestinal tumourigenesis more effectively than either treatment alone (median small intestinal adenoma volume (47 mm^3^ (comb) *vs* 248 mm^3^ (AZ12253801), *P*=0.0003 and 47 mm^3^ (comb) *vs* 123 mm^3^ (gefitinib), *P*=0.0042, Mann–Whitney (two-sided) test).

**Conclusion:**

Our data provide evidence in support of the use of combinatorial therapy, and establishes the need to further define the precise benefit *in vivo*.

Since the discovery of the benefit of EGFR-targeted monoclonal antibody therapy in patients with *KRAS* wild-type metastatic colorectal cancer (Karapetis *et al*, 2008), it has become increasingly important to understand the mechanisms operating in tumours that eventually develop drug resistance. Much recent research has focused on the use of cell culture and xenograft approaches (Guix *et al*, 2008; Haluska *et al*, 2008; Hynes and MacDonald, 2009). Here, we extend that approach to an autochthonous mouse model, namely the *Apc*^*min*/+^ mouse, which develops multiple intestinal adenomas as a result of Wnt pathway activation. The *Apc*^*min*/+^ mouse is of particular relevance as we show that harvested colon adenomas are wild type for *K-ras* and therefore relevant for the study of EGFR-targeted therapy and resistance.

The type 1 insulin-like growth factor receptor (IGF1R) has been implicated in various aspects of tumour development and metastasis (Chitnis *et al*, 2008; Pollak, 2008) with overexpression of IGF1R and its ligands IGF1 and IGF2, a common finding in malignant disease (Furstenberger and Senn, 2002). It has been suggested that signalling through type 1 IGF1Rs may confer resistance to EGF receptor family blockade (Lu *et al*, 2001; Jones *et al*, 2004; Buck *et al*, 2008; Guix *et al*, 2008) and is activated in response to chemotherapy for treatment of colorectal cancer (Dallas *et al*, 2009). Interestingly, resistance to IGF1R-targeted therapies has been demonstrated in a reciprocal fashion with upregulation of EGFR and its ligands suggesting that EGFR pathway activation may be an alternative route for growth signals to be transmitted in presence of inhibition of IGF1R signalling (Haluska *et al*, 2008; Huang *et al*, 2009). Clinical evidence supporting IGF1R as a treatment target for Ewing's sarcoma, adrenocortical carcinoma and non-small cell carcinoma (Carden *et al*, 2009) highlights the need to understand the resistance pathways, which will emerge in response to antagonism of the IGF1 receptor. Given the possible bidirectional cross-communication between EGFR and IGF1R pathways it is not surprising that research has been directed towards combination-targeted therapy against both these pathways.

We show here that acute administration of gefitinib inhibits EGFR signalling while also activating the IGF1R pathway in adenomas developing in the *Apc*^*min*/+^ mouse, an *in vivo* physiologically relevant model of intestinal tumourigenesis. This led us to test the efficacy of combined EGFR/IGF1R antagonism compared with monotherapy with each drug. This is the first long-term study of these treatment approaches in an autochthonous model of *K-ras* wild type intestinal tumourigenesis to examine tumour phenotypic change, adding significantly to the body of evidence supporting the importance of EGFR and IGF1R interactions.

## Materials and methods

### Animals

All experimental male *Apc*^*min*/+^ mice were in-bred on a C57BL/6 background for at least 12 generations and housed in cages (max three per cage) with a 12 h day/light cycle. Mice received expanded RM(3) diet (Special Diet Services, Essex, UK) and tap water *ad libitum*. Weaning took place at ∼4 weeks of age. Procedures involving animals and their welfare were conducted in accordance with the institutional guidelines that comply with United Kingdom national policies (Animals (Scientific Procedures) Act 1986). The *Apc*^*min*/+^ mice were genotyped at 6–8 weeks of age using tail tip material. Further details are provided in Supplementary Information.

### Pyrosequencing for *K-ras* mutations

To detect murine *K-ras* mutations in codons 12/13 and 61 pyrosequencing technology was employed. Codons 12/13 and 61 were initially amplified by PCR using the following primer sequences: codons 12/13 forward (5′-GGCCTGCTGAAAATGACTGA-3′) and reverse (5′-CGCAGACTGTAGAGCAGCGTTAC-3′), codon 61 forward (5′-TGTTTCTCCCTTCTCAGGACTC-3′) and reverse (5′-AGAAAGCCCTCCCCAGTTC-3′). The sequencing primers for codon 12/13 were 5′-CTTGTGGTGGTTGGAG-3′ and codon 61 5′-GGATATTCTCGACACAGC-3′. Pyrosequencing was semi-automated using the Pyromark ID Qiagen System (West Sussex, UK) and both assays were designed to detect all possible mutations in the codons examined. See Supplementary Information for further detail.

### Allelic discrimination assay for *B-raf V600E* mutation

Amplification of a specific sequence of target DNA within the *B-raf* gene was achieved using forward (5′-TTCATGAAGACCTCACAGTAAAAATAGG-3′) and reverse (5′-TCGATGGAGTGGGTCCCA-3′) primer sequences. Thereafter TaqMan probes for hybridisation to the target sequence within the PCR product were used to detect wild-type or V600E *B-raf* mutation. The probe sequences were *B-raf* wild type (VIC-5′-AGCTACAGTGAAATC-3′), *B-raf* V600E (6FAM-5′-CTACAGAGAAATCTC-3′). See Supplementary Methods for further detail.

### Materials for injection

Small molecular tyrosine kinase inhibitors of the epidermal growth factor and type 1 IGF receptors, gefitinib and AZ12253801, respectively, were made available by AstraZeneca (Cheshire, UK). Gefitinib was suspended in purite water containing 0.5% Tween 80 for acute experiments (or 1% Tween 80 for chronic exposure experiments or as indicated) and injected via the intra-peritoneal (i.p.) route at a dose of 75 mg kg^−1^. The AZ12253801 was suspended in purite water containing 1% Tween 80 and dosed at 12.5 mg kg^−1^ i.p. once daily. Bromodeoxyuridine (Brdu, GE Healthcare, Amersham, UK) S phase cell labelling experiments were undertaken using a single i.p. injection of 200 *μ*l (3 mg ml^−1^) 2 h before death.

### Animal dissection/tissue preparation

Male *Apc*^*min*/+^ mice were culled by cervical dislocation in acute experiments 4 h following drug administration or in chronic experiments when mice had reached the defined cohort age. Following a midline excision, small and large intestines were identified, removed and flushed using cold tap water. The large bowel was incised along the mesenteric border to open the luminal surface to permit resection of colon adenomas proximal to any adenomas related to rectal prolapse. Colon adenomas were immediately placed in RNAlater (Applied Biosystems, Austin, TX, USA) or snap frozen in liquid nitrogen and stored at −80°C. Small intestines were sectioned in the transverse plane at three 1 cm intervals, 10 cm distal to the gastro-duodenal junction and placed in a fixing parcel of 3 m surgical tape before overnight fixation in 10% formalin. The remaining small intestine and large intestine were placed on Whatman paper and sectioned along the mesenteric border to open the luminal surface to facilitate fixation using methacarn solution (methanol: chloroform: acetic acid; 4 : 2 : 1). Small and large intestine adenoma counts, size measurements and location were recorded before rolling gut tissue into a Swiss roll, securing and placing in 96% ethanol. Immunohistochemistry and western blotting techniques are included in Supplementary Materials.

### Acute drug exposure experiments in *Apc*^*min*/+^ mice

To assess the early (4 h) pharmaco-dynamic effects of each drug on cell death, cell proliferation, immune-reactive staining and protein changes, small cohorts of three mice (unless otherwise stated) were used to obtain appropriate adenoma tissue. Mice with an intestinal adenoma burden (i.e., pale feet) were exposed to agents. Phenotypic scoring of adenomas is described in Supplementary Materials.

### Long-term drug exposure in *Apc*^*min*/+^ mice

A minimum of 15 *Apc*^*min*/+^ mice (range 15–17) were used in each chronic drug exposure cohort. Mice started treatment at a median age of 56 days for all treatments. Each mouse (except vehicle controls, cohort D) was exposed to daily i.p. gefitinib (75 mg kg^−1^) for 8 weeks to permit the possible development of gefitinib-resistant intestinal adenoma clones. Cohort (A) continued daily gefitinib dosed at 75 mg kg^−1^; cohort (B) continued daily gefitinib 75 mg kg^−1^ in addition to daily i.p. AZ12553801 dosed at 12.5 mg kg^−1^; cohort (C) stopped gefitinib following 8 weeks treatment and started AZ12553801 as a single agent dosed at 12.5 mg kg^−1^ o.d.; and finally cohort (D) received continuous daily i.p. injections of vehicle control 1% Tween 80 from the outset, dosed according to body weight. Cohorts A–C were aged to 200 days and then culled. The vehicle control cohort (D) became symptomatic of adenoma burden, (evidenced by pale feet, hunching, piloerection or weight loss ⩾10% starting body weight) and was culled at day 150. Adenomas in the small and large intestine were counted and measured to enable the calculation of total adenoma volume. Adenoma location was also recorded.

### Statistical analysis

The Mann–Whitney (two-sided) statistics was used to detect significant differences in adenoma numbers and total adenoma volumes following chronic drug exposure. Box plots of small and large intestinal numbers and adenoma volumes were generated using Minitab software v15 (Minitab Ltd, Coventry, UK). Error bars on charts represent ±1 s.d. from the mean value and non-overlapping. *P*-values of ⩽0.05 were accepted as statistically significant.

## Results

### *Apc*^*min*/+^ colon adenomas do not harbour mutations in *K-ras* or *B-raf* alleles

To determine *K-Ras* and *B-raf* status in adenomas developing in *Apc*^*min*/+^ mice, we performed *K-ras* pyrosequencing for known mutations in codons 12, 13 and 61 and allelic discrimination assays for *B-raf* V600E, which failed to identify mutations in genomic DNA from 30 individual *Apc*^*min*/+^ colon adenomas harvested from 29 mice (data not shown).

### Acute *in vivo* EGFR inhibition suppresses EGFR signalling, induces anti-tumour pharmacodynamic changes and activates the IGF1R receptor in *Apc*^*min*/+^ colon adenomas

**Figure 1:**
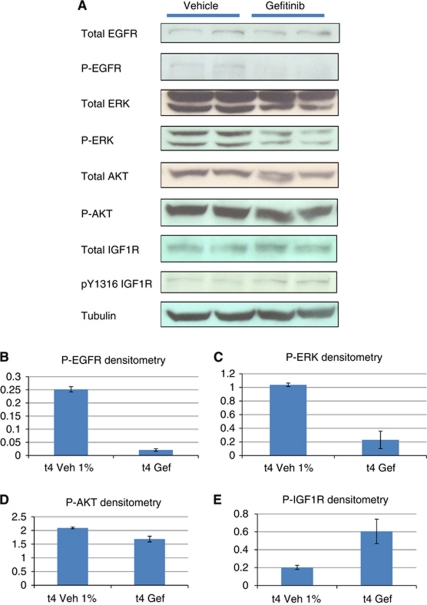
(**A**) Western blot to demonstrate the immediate *in vivo* effect of gefitinib 75 mg kg^−1^ on downstream EGFR signalling in *Apc*^*min*/+^ colon adenomas. A 30-*μ*g pooled colon adenoma protein sample was loaded into each well in duplicate. Each loaded sample comprised equivalent amounts of protein (30 *μ*g) from individual colon adenomas harvested from male *Apc*^*min*/+^ mice exposed to either vehicle (1% Tween 80) or gefitinib. Colon adenomas were obtained 4 h post dosing when animals were culled. A total of 26 colon adenomas from three gefitinib-treated mice and 19 colon adenomas from three vehicle-treated mice were pooled. Loading controls were either *β* actin or tubulin. (**B**–**E**) Densitometry of total phospho-protein levels normalised relative to loading control with value ranges indicated by error bars.

**Figure 2:**
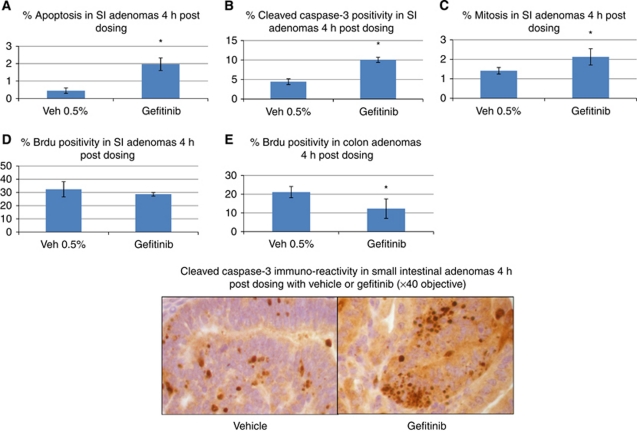
The acute pharmacodynamic effects of gefitinib 75 mg kg^−1^ (i.p.) compared with vehicle control (0.5% Tween 80) in *Apc*^*min*/+^ small intestinal (SI) adenomas (**A**–**D**) and colon adenomas (**E**) 4 h post dosing. (**A**) Gefitinib-induced apoptosis in SI adenomas (0.45%±0.15 (Veh) *vs* 1.96%±0.36 (Gef), *P*=0.04) and (**B**) cleaved caspase-3 staining (4.4%±0.7 (Veh) *vs* 10.1%±0.7 (Gef), *P*=0.04). (**C**) Increased SI mitotic scoring (1.4±0.2 (Veh) *vs* 2.1%±0.4 (Gef), *P*=0.04) with (**D**) unchanged Brdu labelling (32.4%±5.5 (Veh) *vs* 28.6%±1.4 (Gef), *P*=0.3) following gefitinib exposure. (**E**) Colon adenoma Brdu labelling is reduced following gefitinib exposure (21.1%±3 (Veh) *vs* 12.3%±5.2 (Gef), *P*=0.04). A minimum of three tumours were scored from each animal with three mice for each experimental treatment (^*^P=0.04).

We next tested the effects of acute EGFR blockade using gefitinib in adenoma bearing *Apc*^*min*/+^ mice. Four hours following exposure to 75 mg kg^−1^ gefitinib we observed reduced levels of phosphorylated EGFR in colon adenomas and suppressed downstream phosphorylation of ERK and AKT (Figure 1A–D). We also observed activation of the IGF1 receptor as indicated by increased levels of IGF1R phosphorylation at tyrosine 1316 (Figure 1E). Phenotypically, gefitinib exposure led to increased levels of apoptosis as scored by both H and E staining and the presence of cleaved caspase-3. In both the small and large bowel there was evidence of perturbed cell cycling. In small intestinal adenomas, levels of Brdu labelling were unchanged at 4 h. However, there was an increase in the number of mitotic figures, suggestive of an M phase arrest (Figure 2A–D). In colonic adenomas, we observed a direct reduction in Brdu labelling (Figure 2E), although there was no change in mitotic counts (data not shown).

In light of the ability of EGFR inhibition to suppress downstream signalling in intestinal adenomas, induce favourable pharmacodynamic change and increase IGF1 receptor signalling, we next investigated the long-term effects of single agent EGFR antagonism, single agent IGF1R inhibition and combination EGFR and IGF1R inhibition.

### Chronic EGFR inhibition suppresses *Apc*^*min*/+^ intestinal tumorigenesis

**Figure 3:**
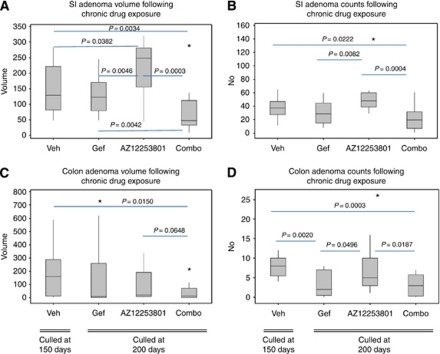
Box plots illustrating the treatment effects of EGFR inhibition (gefitinib), IGF1R inhibition (AZ12253801), vehicle (1% Tween 80) and combined EGFR/IGF1R inhibition on *Apc*^*min*/+^ mice. Cohorts of male *Apc*^*min*/+^ mice (*n*=15–17) were treated with gefitinib for a period of 8 weeks and subsequently exposed to single (either gefitinib or AZ12253801) or combined (gefitinib and AZ12253801) therapy. Treated mice were culled at 200 days. Vehicle-treated mice were not exposed to gefitinib and culled at 150 days due to symptomatic disease. (**A**–**D**) Box plots to show adenoma number and adenoma volumes at death for both the small intestine and colon with associated *P*-values (two-sided Mann–Whitney test). A total of 14 mice received daily vehicle (1% Tween 80), 17 gefitinib (75 mg kg^−1^), 15 AZ12253801 (12.5 mg kg^−1^) and 16 combined treatment with gefitinib and AZ12253801. Abbreviations: AZ12253801=IGF1R inhibitor; Gef=gefitinib; Combo=Gef/AZ12253801 combination; SI=small intestine; Veh=vehicle. A *P*-value of ⩽0.05 was considered significant (asterisk represents an outlying value).

The *Apc*^*min*/+^ mice exposed daily to 75 mg kg^−1^ gefitinib were culled at 200 days. Vehicle-treated animals developed an overt phenotype before reaching 200 days and were culled at 150 days. Median colon adenoma numbers at death were significantly reduced in gefitinib-treated animals compared with vehicle treatment (2 *vs* 8 colon adenomas, *P*=0.002; Figure 3D). As no difference was observed in colon adenoma volume between gefitinib and vehicle treatments (Figure 3C), this suggests that the reduced number of colon adenomas in gefitinib-treated mice grew to a larger size, presumably as a direct consequence of enhanced longevity. Despite the suppression of disease symptoms and consequent increased survival, no differences were observed in the median number of small intestinal adenomas (37.5 *vs* 29, *P*=0.2664; Figure 3B) or small intestinal adenoma volumes (128 *vs* 123 mm^3^, *P*=0.4998; Figure 3A), indicating that following gefitinib exposure, a longer period of time is required to develop the same adenoma burden. We therefore interpret this data to argue that gefitinib exposure suppresses adenoma development in both small and large intestine.

### Chronic IGF1R inhibition fails to inhibit *Apc*^*min*/+^ intestinal tumourigensis

The *Apc*^*min*/+^ mice were initially treated daily with 75 mg kg^−1^ gefitinib for 8 weeks, and were subsequently exposed to a daily dose of 12.5 mg kg^−1^ AZ12253801. Mice were then killed and analysed at 200 days of age. There was an increase in median small intestine adenoma volume relative to the vehicle cohort culled at 150 days (128.5 *vs* 248 mm^3^, *P*=0.0382; Figure 3A). There was no difference in small intestinal adenoma count (37.5 *vs* 48, *P*=0.0668; Figure 3B), indicating that adenomas grew to a larger size in the AZ12253801-treated cohort. There were also no differences in either median colon adenoma volume (158.5 *vs* 19.5 mm^3^, *P*=0.2280; Figure 3C) or colon adenoma number. These data suggest that exposure to AZ12253801 suppressed adenoma development in the large intestine, as adenoma burden remained the same, despite increased cohort time. Notably, there was a higher small intestinal adenoma burden, presumably as a direct consequence of increased longevity.

### Chronic EGFR/IGF1R inhibition maximally suppresses *Apc*^*min*/+^ small intestinal tumourigenesis

The *Apc*^*min*/+^ mice initially treated with daily 75 mg kg^−1^ gefitinib for 8 weeks, were subsequently treated with daily gefitinib in combination with 12.5 mg kg^−1^ AZ1225380. At 200 days, combined treatment resulted in significantly reduced small and large intestinal tumourigenesis relative to vehicle as measured by both adenoma number and volume (median number small intestinal adenomas 37.5 *vs* 20, *P*=0.0222; median number colon adenomas 8 *vs* 3, *P*=0.0003; median small intestine adenoma volume 128.5 *vs* 47 mm^3^, *P*=0.0034; median colon adenoma volume 158.5 *vs* 10.5 mm^3^, *P*=0.0150; Figure 3A–D). The combination of EGFR/IGF1R blockade therefore reduces both small and large intestinal tumourigenesis. When comparison is made between the three chronic treatment regimes at 200 days, combination treatment was observed to reduce the median small intestinal adenoma number compared with AZ12253801 (20 (combo) *vs* 48 (AZ12253801), *P*=0.0004) but not against gefitinib (20 (combo) *vs* 29 (gefitinib), *P*=0.22; Figure 3B). Critically, combination treatment markedly reduced the median small intestinal adenoma volume compared with either single treatment (47 mm^3^ (comb) *vs* 248 mm^3^ (AZ12253801), *P*=0.0003 and 47 mm^3^ (combo) *vs* 123 mm^3^ (gefitinib), *P*=0.0042; Figure 3A). In the large intestine, combined therapy reduced colon adenoma number compared with AZ12253801 treatment, but not against gefitinib (3 (combo) *vs* 5 (AZ12253801), *P*=0.0187 and 3 (combo) *vs* 2 (gefitinib), *P*=0.718; Figure 3D). No differences were observed in median colon adenoma adenoma volume between the treatments (10.5 mm^3^ (combo) *vs* 19.5 mm^3^ (AZ12253801), *P*=0.0648 and 10.5 mm^3^ (combo) *vs* 8 mm^3^ (gefitinib), *P*=0.482; Figure 3C).

### Acute phenotypic changes induced in small intestinal treated with EGFR/IGF1R inhibition

We next sought to dissect the mechanisms of response to these agents in terms of the acute effects upon *Apc*^*min*/+^ intestinal adenomas with respect to cell death and proliferation rates. Following 4 h exposure of *Apc*^*min*/+^ mice to combined EGFR/IGF1R antagonism, there is a reduction in small intestinal adenoma mitotic scoring (0.4%±0.3 (combo) *vs* 2.1%±0.4 (gefitinib), *P*=0.04) and Brdu cell labelling (16.9%±4 (combo) *vs* 28.6%±1.4 (gefitinib), *P*=0.04) compared with gefitinib, without the detection of an increased level of small intestinal adenoma apoptosis (1.4%±0.5 (combo) *vs* 1.9±0.4 (gefitinib)) or caspase-3 scoring (5.2%±3.8 (combo) *vs* 10.1%±0.7 (gefitinib).

### The molecular effects of acute IGF1R inhibition

**Figure 4:**
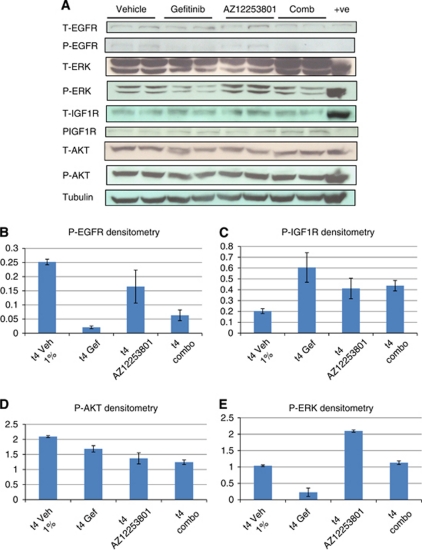
(**A**) Western blotting to demonstrate the immediate molecular signalling changes induced in *Apc*^*min*/+^ colon adenomas 4 h post single dosing with vehicle (1% Tween 80), gefitinib 75 mg kg^−1^ o.d., AZ12253801 12.5 mg kg^−1^ o.d. and combined gefitinib and AZ12253801. A volume of 30 *μ*g pooled colon adenoma protein samples were loaded into wells in duplicate with a single positive control. Each loaded sample comprised equivalent amounts of protein (30 *μ*g) from individual colon adenomas harvested from three male *Apc*^*min*/+^ mice exposed to either vehicle, gefitinib, AZ12253801 or combined geftitinb and AZ12253801 for 4 h. A total of 19 colon adenomas were pooled from vehicle-treated mice, 26 colon adenomas from gefitinib-treated mice, 9 colon adenomas from AZ12253801-treated mice and 17 colon adenomas from combined-treated mice. Loading controls were either *β* actin or tubulin. (**B**–**E**) Densitometry readings for each phosphorylated protein were normalised relative to loading controls. Error bars indicate value ranges. Abbreviations: AZ12253801=IGF1R inhibitor; combo=combined EGFR/IGF1R inhibition; Gef=gefitinib; Veh=vehicle.

The immediate effect of gefitinib exposure is to reduce phosphorylation of EGFR, ERK and AKT with reciprocal phosphorylation of the IGF1R (Figure 4). We next investigated the molecular changes occurring 4 h following IGF1R inhibition. Inhibition of IGF1R signalling by AZ12253801 alone results in an expected reduction in phosphorylation of AKT (Figure 4A and D) and an apparent paradoxical ‘rebound’ increased phosphorylation of the IGF1R (Figure 4A and C). In the context of a reduced level of EGFR phosphorylation (Figure 4A and B), AZ12253801 also induced phospho-ERK1/2 signalling (Figure 4A and E). Thus we can hypothesise that inhibition of IGF1R initially suppresses downstream signalling as evidence by the reduction in phospho-AKT, but this change leads to a loss of feedback upstream resulting in increased IGF1R phosphorylation. Such rebound activation of the IGF1R pathway following IGF1R inhibition may explain the increased level of ERK phosphorylation or alternatively reflect altered EGFR trafficking.

### The molecular effects of acute combination therapy

In view of the signalling changes described for EGFR and IGF1R inhibition alone we anticipated that combined EGFR/IGF1R inhibition would produce either competing or additive molecular effects 4 h after exposure. The former is demonstrated for both EGFR and ERK phosphorylation (Figure 4B and E) where the level of activity is between that for each agent alone. For phospho-AKT suppression, we observed an additive effect compared with either treatment in isolation (Figure 4D). For phospho-IGF1R, combination therapy resulted in either similar (IGF1R inhibition) or reduced (EGFR inhibition) levels compared with individual treatments (Figure 4C).

### The molecular effects of chronic gefitinib or AZ12253801 exposure

Finally, we examined signal transduction pathway changes associated with chronic administration to determine potential tumour resistance mechanisms and to ask if the acute signalling pathway changes bear any direct relevance to those signalling pathways altered following chronic exposure.

**Figure 5:**
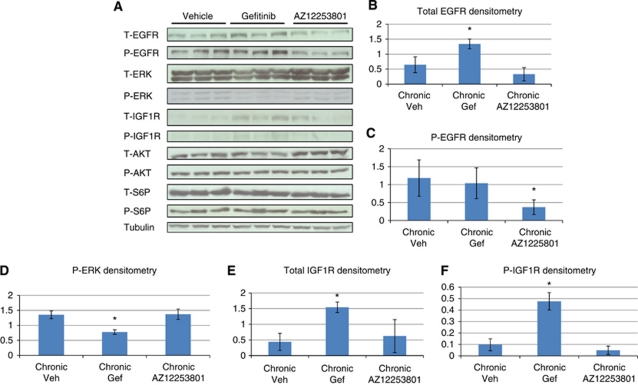
(**A**) Western blot to demonstrate the molecular signalling activity in colon adenomas obtained from male *Apc*^*min*/+^ mice exposed to long-term vehicle, gefitinib 75 mg kg^−1^ o.d. or AZ12253801 12.5 mg kg^−1^ o.d. treatment (**A**). Colon adenomas were obtained at the time of death (150 days for vehicle and 200 days for mice receiving treatment). A volume of 30 *μ*g pooled colon adenoma protein samples were loaded into wells in triplicate. Each loaded sample comprised equivalent amounts of protein (30 *μ*g) from individual colon adenomas harvested from three male *Apc*^*min*/+^ mice except for vehicle treatment where two mice were used. In total seven colon adenomas were pooled from vehicle-treated mice, eight from gefitinib-treated mice and 13 from AZ12253801-treated mice. Loading control was tubulin. (**B**–**F**) Densitometry values for respective total and phosphorylated proteins. Phosphorylated proteins were normalised relative to loading control reflecting total levels of protein phosphorylation. Error bars represent ±1 s.d., ^*^P=0.04 (Mann–Whitney test).

Chronic treatment with gefitinib (relative to vehicle) results in increased protein levels of total EGFR, total IGF1R and phospho-IGF1R (Figure 5A, B, E and F). Given that levels of both EGFR and ERK phosphorylation (Figure 5A, C and D) are not elevated, this argues that IGF1R activity may be driving resistance to therapy. However, despite evidence of activation of the IGF1 receptor, phosphorylation of the downstream effectors usually associated with this pathway (AKT and S6 ribosomal protein densitometry data are not shown) is not seen. This suggests that, in the setting of chronic blockade of EGFR, activation of the IGF1R pathway is mediating adenoma growth through alternative, unidentified mechanisms. Furthermore, the observation that acute gefitinib exposure induced activation of the IGF1R at 4 h highlights that resistance mechanisms are initiated early in a treatment schedule and that they can be predicted by this method.

The only change observed following chronic AZ12253801 was a reduction in phospho-EGFR levels relative to vehicle (Figure 5). Given the suppressed level of phospho-EGFR, the maintenance of phospho-ERK (Figure 5C and D) is presumed to be via an EGFR independent mechanism. Chronic IGF1R inhibition therefore results in similar pathway dynamics to acute IGF1R inhibition, where we also observed suppression of phospho-EGFR. If the level of phospho-ERK is crucial in driving IGF1R resistant adenomas, then the acute data showing an increase in phospho-ERK potentially reflects a mechanism of resistance. This raises the possibility that IGF1R inhibitor resistance may be overcome by combined IGF1R and MEK inhibition.

## Discussion

In this paper we have described the acute and chronic effects of inhibiting EGFR and IGF1 alone and in combination in the *Apc*^*min*/+^ mouse. Given that *K-RAS and B-RAF* wild type status is predictive of EGFR-targeted response in patents (Di Nicolantonio *et al*, 2008; Karapetis *et al*, 2008), we first determined *K-ras* and *B-raf* status in adenomas developing in the *Apc*^*min*/+^ mouse. The observation that these adenomas remain wild type for *K-ras* and *B-raf* underscores the relevance of this model for studying EGFR blockade. In terms of responsiveness to agents that target EGFR and IGF1R, there is increasing evidence for cross-communication between these two pathways from both cell line and xenograft studies (Buck *et al*, 2008; Guix *et al*, 2008; Haluska *et al*, 2008; Desbois-Mouthon *et al*, 2009; Huang *et al*, 2009; Kaulfuss *et al*, 2009). Here we have now extended this analysis to an autochthonous *in vivo* animal model.

Acute exposure of *Apc*^*min*/+^ mice to gefitinib suppressed EGFR, ERK and AKT phosphorylation in keeping with its known action as an inhibitor of EGFR tyrosine kinase activity (Wakeling *et al*, 2002). This, combined with published evidence of the importance of EGFR signalling in *Apc*^*min*/+^ mouse intestinal adenomas (Moran *et al*, 2004) including genetic manipulation studies with hypomorphic EGFR alleles (EGFR^wa2^ allele) and pharmacologic manipulation (Roberts *et al*, 2002), supports a role for EGFR in intestinal adenoma development. Short-term exposure to gefitinib led to increased apoptosis and mitotic blockade in small intestinal adenomas and reduced cell cycling in colon adenomas. We also observed increased IGF1R phosphorylation. These observations predict that long-term exposure to gefitinib will improve survival by delaying the development of intestinal adenomas and implicit in this the notion that gefitinib resistance may develop, possibly through deregulation of pathways such as IGF1R. This early observation indicated to us that this model would be useful to test the effect of IGF1R antagonism alone and in combination with gefitinib.

Chronic administration of gefitinib reduced intestinal tumourigenesis as a consequence of cell cycle inhibition. This observation of perturbed cell cycling supports an earlier report of gefitinib reducing proliferation, the number of aberrant crypt foci and colonic microadenomas in an azoxymethane model of colonic carcinogenesis (Fichera *et al*, 2007). We have confirmed the significance of early gefitinib induced IGF1R signalling in colon adenomas in relation to resistance as IGF1R and IGF1R phosphorylation are increased at the protein level in chronic gefitinib exposed resistant adenomas. Interestingly phospho-AKT and phospho-S6P were not activated, suggesting an alternate pathway is responsible for signal transduction promoting adenoma growth. It appears that adenoma cells in an attempt to overcome EGFR blockade also increase EGFR protein but fail to increase activation of EGFR signalling as shown by an absence of change in EGFR phosphorylation and reduced ERK phosphorylation. Previous work has shown that inhibition of EGFR-ERK pathway activity by EGFR blockade enhances IRS1 interaction with PI3K-AKT signalling (Buck *et al*, 2008). This together with our finding of increased IGF1R activity following EGFR blockade led us to expect increased AKT phosphorylation, however, we failed to demonstrate this *in vivo* following chronic gefitinib treatment.

The acute increased IGF1R activity in colon adenomas following exposure to EGFR blockade raises the possibility of testing tumour specimens or circulating tumour cells (de Bono *et al*, 2007) for their initial response to drug. Such responsiveness could then be considered in determining therapeutic combinations. To address this approach, we determined if the acute activity in IGF1R predicted improved outcome (in terms of anti-adenoma effect) when IGF1R inhibition was combined with EGFR blockade. We have demonstrated suppression of intestinal tumourigenesis with combination treatment relative to vehicle as evidence by an almost three-fold reduction of median small intestinal adenoma volume and 16-fold reduction of median colon adenoma volume. The reduction in small intestinal adenoma volume remains when compared with gefitinib treatment alone reinforcing the potential therapeutic advantage of adding IGF1R blockade to gefitinib.

In terms of understanding the mechanism of the adenoma suppression with combined EGFR/IGF1R treatment, we undertook protein analysis for 4 h following EGFR and IGF1R blockade alone and in combination. We anticipated demonstrating maximal suppression of AKT phosphorylation for the combination against both drug comparisons (Buck *et al*, 2008; Guix *et al*, 2008), however, we only found suppression of tumoural AKT signalling for combination against vehicle and gefitinib treatments (Figure 4D), but not IGF1R inhibition. Expectation of maximal suppression of AKT signalling for the combination does assume a simple additive relationship in terms of the effects seen by either drug in isolation, and it is possible that in combination the relationship between the pathways is more complicated such that at the level of AKT, pathway inhibition and loss of negative feedback does not equate with an additive outcome *in vivo* as expected. Therefore in terms of understanding the signalling responsible for combinatorial adenoma suppression we conclude that relative to vehicle, a reduction in EGFR and AKT phosphorylation appears to be important. At the 4 h timepoint chosen, we have been unable to clearly dissect the anti-tumour mechanism of dual therapy in terms of cell death/turnover. Previous reports of enhanced apoptosis with dual blockade of EGF/IGF1 receptors have been published with (Guix *et al*, 2008) or without increased anti-proliferative effects (Buck *et al*, 2008; Haluska *et al*, 2008).

The IGF1R receptor has been implicated in the development of tumours in various settings (Pollak, 2008) and we therefore anticipated that inhibition of the IGF1R would be of potential therapeutic significance in this model. We observed that exposure to chronic IGF1R inhibition may delay small intestinal adenoma growth, whereas combined IGF1R/EGFR blockade suppresses growth relative to single agent treatments. This suggests continued EGFR inhibition is required to promote IGF1R adenoma expression and the target of AZ12253801, to elicit an anti-adenoma response. In demonstrating EGFR blockade enhanced IGF1R activity we have provided support for the *in vivo* proof of concept that IGF1R-targeted therapy may induce an anti-adenoma effect in adenomas expressing high receptor levels driven by continued EGFR blockade, and is a potential predictive biomarker (Zha *et al*, 2009).

Signalling pathway changes in response to acute IGF1R inhibition (Figure 4) included an expected reduction in AKT phosphorylation but also a paradoxical increase in IGF1R phosphorylation along with an increased level of ERK phosphorylation despite a reduction in EGFR activity. We suggest that AZ12253801 initially suppresses IGF1R phosphorylation leading to the observed reduction in AKT activity, with this itself triggering feedback leading to increased IGF1R phosphorylation. This ‘rebound’ activity in IGF1R signalling may account for the marked increase in ERK phosphorylation. Alternatively, the increased ERK activity may be consequent upon subtle changes in EGFR trafficking or secondary to suppression of cytoplasmic phosphatases DUSP 6, 7 or 9 (Owens and Keyse, 2007).

Taken together, we speculate that the predominant initial response to AZ12253801 is suppression of AKT signalling, followed by a switch to predominant ERK signally resulting in loss of response and the observed increased adenoma growth. If this hypothesis is correct it may be possible to suppress or prevent tumour resistance to IGF1R blockade by inhibiting ERK activity; indeed we do see partial inhibition of ERK phosphorylation by adding gefitinib to IGF1R inhibition (Figure 4E). Such incomplete ERK inhibition may explain why combination with EGFR blockade therapy ultimately fails, and also suggests that more potent suppression of ERK activity for example with a MEK inhibitor in the context of IGF1R inhibition may be a preferred combination (Buck *et al*, 2008).

Chronic exposure to IGF1R inhibition also reduces EGFR phosphorylation. Recent studies support a role for EGFR family members in resistance to IGF1R inhibitors (Haluska *et al*, 2008; Desbois-Mouthon *et al*, 2009; Huang *et al*, 2009) and suggest that at least in the *Apc*^*min*/+^ mouse model the response to chronic IGF1R therapy does not involve increased activity in EGFR. The mechanism of reduced EGFR phosphorylation is unclear.

In summary we have shown that, in *Apc*^*min*/+^ intestinal adenomas, acute EGFR blockade by gefitinib reduces EGFR signalling, but activates IGF1R, a potential resistance pathway. Chronic monotherapy against either EGFR or IGF1R influences adenoma growth, but combination EGFR/IGF1R blockade produced the most effective adenoma suppression. Our data therefore support the concept of EGFR resistance mediated through induced IGF1R signalling supporting the need for further enquiry regarding the potential utility of combinatorial therapy.
