# Supplementary material for: Dual inhibition of epidermal growth factor and insulin-like 1 growth factor receptors reduce intestinal adenoma burden in the Apcmin/+ mouse
Source: Br J Cancer. 2011 Aug 2;105(5):649–57. doi: 10.1038/bjc.2011.291 (PMC3188935; doi:10.1038/bjc.2011.291)
Supplement: Supplementary Information [file bjc2011291x1.doc]

**Supplementary information**

**Animal genotyping**

Genomic DNA was extracted using Puregene™ cell lysis solution containing 2% proteinase K. Samples were incubated overnight at 37^0^C or at 55^0^C for three hours. Puregene™ protein precipitating solution (Gentra systems) was added to the tube and inverted 3-4 times and centrifuged at full speed for 10min. Supernatants were placed in 500μl of isopropanol and inverted 3-4 times followed by centrifugation at full speed for 15min. Supernatant was removed leaving each DNA pellet which was air dried for 1 hour prior to the addition of 500μl of nuclease free water. Mice were genotyped for the *Apc* gene using TCT CGT TCT GAG AAA GAC AGA AGC T forward and TGA TAC TTC TTC CAA AGC TTT GGC TAT reverse primer sequences and the modifier of min locus (*Mom*) using GCT TGC TTT AGG AGT GTG CC forward and TAT TTG CTC TCC ATT TCC CC reverse primers. Each primer (0.1μl of 100uM) was added to 2.5μl of genomic DNA and mixed with 0.4μl of Pic Taq DNA polymerase (CRUK); 5μl of buffer (10mM TRIS, 50mM KCl, pH 8.3); 5μl MgCl_2_ (25mM) and 0.4μl dNTPs (25Mm of each dNTP, Invitrogen) . PCR conditions were 94°C for 2min; (94°C for 1min; 60°C for 1min; 72°C for 1min) for 30cycles followed by 72°C for 10min. The *Apc* gene primer product underwent Hind III digest (Promega) and was run on a 4% agarose gel for 30min at 120volts giving a 144bp product for the Min allele and 123bp for wild type alleles. The *Mom* PCR reaction produced a 194bp product on a 2% agarose gel. All experimental mice were re-genotyped at death to corroborate initial genotyping.

***K-ras* pyrosequencing**

PCR reaction mix contained:-

| Reaction mix | Stock | Final | 1x mix (μl) |
| --- | --- | --- | --- |
| Megamix Gold (Microzone Ltd) | - | - | 12.5 |
| Forward primer | 100pm/μl | 10pm/μl | 0.25 |
| Reverse primer | 100pm/μl | 10pm/μl | 0.25 |
| dH_2_O |  |  | 10 |
| DNA | 5/10ng/μl |  | 2 |
| Total PCR volume |  |  | 25 |

PCR reaction conditions:-

|  | Temp. [^o^C] | Time | Step |
| --- | --- | --- | --- |
| Initial denaturation | 95 | 10 min | 1 |
| Denaturation | 95 | 30 sec | 2 |
| Annealing | 60 | 30 sec | 3 |
| Synthesis | 72 | 30 sec | 4 (return 2 x40) |
| Final synthesis | 72 | 10 min | 5 |

***B-raf* allelic discrimination assay**

PCR reaction mix:-

| Reagent | 1x |
| --- | --- |
| TaqMan (2x) | 12.5 |
| Primers F/R (10μM) | 2.5 |
| Probes-Norm/V600E (5μM) | 1 |
| DNA | 5 |
| dH_2_O | 4 |

| Reaction conditions | | | |
| --- | --- | --- | --- |
|  | Temp [^o^C] | Time | Step |
| Initial denaturation | 95 | 10 mins | 1 |
| Denature | 92 | 15 sec | 2 |
| Anneal/extend | 60 | 1 min | 3 (return 2 x40) |

Data collection for the assay was recovered during the annealing and extension phase.

**Immuno-histochemistry**

Histological sections 5μ thick were obtained from formalin fixed and paraffin embedded tissues and rehydrated by sequential bathing in 100% xylene (5 mins x2), 100% ethanol (2 mins), 95% ethanol (2 mins x2), 70% ethanol (2 mins) and distilled water prior staining. Primary antibodies included rabbit anti cleaved caspase-3 (CST#9661 1/200 dilution) and mouse anti Brdu (Becton Dickinson Immunocytometry Systems, N347580; 1/150 dilution). Antigen unmasking was performed by placing slides in citrate buffer at boiling point for 20 mins. Biotinylated goat anti-rabbit secondary antibody (DAKO North America, Inc.) and the Vectastain Elite ABC kit (PK-6100 series, Vector laboratories, Inc., CA) was used for cleaved caspase-3 and anti-mouse HRP labelled polymer (Envision+ kit, DAKO) for Brdu immuno-staining. Visualisation was facilitated by DAB+Chromogen Envision™ system (DAKO).

**Apoptosis and cell proliferation scoring**

Haematoxylin and eosin or alternatively stained sections of tumour tissue using anti-cleaved caspase-3 or anti-Brdu antibodies were viewed using x40 lens (Olympus BX41 microscope, Japan) with Olympus colour view software imaging and U-CMAD3 camera. Representative areas of tumour tissue aided by use of analysis imaging software were scored for the average number of epithelial cells per x40 field of view using 3 separate regions of a tumour. Phenotypic scoring (apoptotic bodies, cell mitoses, positive immune-reactive staining for cleaved capsase-3 or Brdu positivity) was performed by one person (PS) in a blinded fashion and results expressed as a percentage of the average number of epithelial cells per x40 field of view. For each experimental mouse tumour at least 1000 epithelial cells were counted/3 fields of view and most commonly 3 tumours were scored from each animal. The average values for each phenotypic score were calculated using 3 experimental mice.

**Western blotting.**

Protein was extracted from colon polyps (stored at -80°C) using the illustra™ triple test kit (GE Healthcare, Amersham, UK) by following the manufacturer’s instructions. Protein estimation was enabled using the 2D Quantification Kit (GE Healthcare, Amersham, UK). 30ug samples of polyp protein were pooled from experimentally identical mice exposed to either acute or chronic 1% tween^80^ vehicle, gefitinib, AZ12253801 or combination gefitinib and AZ1225380 at appropriate doses. 30ug protein samples for each experimental condition were loaded into 7 or 9% SDS-PAGE following 95^o^ C incubation for 3 minutes with 5μl Laemelli buffer and 5μl 10% (w/v) SDS. Standard protein markers were used (BioRad™ Precision plus All Blue). Gels were transferred to PVDF membrane over 1.5 hours using methanol based transfer buffer. Membranes were blocked using 5% skimmed milk protein (Marvel) or 5% BSA (Sigma) in 0.1% TBS tween^20^ (sigma) for one hour at room temperature. Primary antibodies Total EGFR CST#2232, 1/1000; Phospho-EGFR (Tyr1068) CST#2243, 1/300; Total ERK CST#9102, 1/1000; Phospho 44/42 MAPK (ERK1/2 Thr202/Tyr204) CST#4376, 1/1000; Total IGF-1Rβ CST#3027, 1/1000; Phospho (Tyr1316) IGF-1R, AZ gift, 1/300; Total AKT CST#9272, 1/1000; Phospho-AKT (Ser473) CST#3787, 1/300; Total S6 Ribosomal Protein CST#2217, 1/1000, Phospho-S6 (Ser240/244) Ribosomal Protein CST#2215, 1/1000 and Tubulin AbCam Ab6160, 1/2000 were incubated overnight at 4°C at indicated dilutions in recommended blocking buffer. HRP-labelled secondary antibodies raised against the source animal primary antibody were incubated at room temperature for 1 hour at a 1:2000 dilution. Membranes were washed 3 times for 5 minutes each using 0.1% TBS tween^20^ following both primary antibody and secondary antibody incubations. ECL™ standard (RPN2109) or ECL™ Plus (RPN2132) western blotting detection reagents (GE Healthcare, Amersham, UK) produced chemi-luminescent light detected by Fuji film and developed using the Xograph imaging system (Compact x4).

**Densitometric analysis**

Western films were scanned and processed using Quantiscan software v1.0 (Biosoft). Discrete blots were analysed after being defined manually and densitometric volumes reported following background subtraction. For each blot a normalised value was calculated with reference to the loading control and subsequent average densitometric values produced. Average densitometric values were obtained for each experimental approach and compared accordingly. Phosphorylated proteins are expressed as the total amount rather than as a proportion of the total protein in question.
